# Indigenous peoples and the COVID-19 pandemic: a systematic scoping review

**DOI:** 10.1088/1748-9326/acb804

**Published:** 2023-02-13

**Authors:** Kerrie Pickering, Eranga K Galappaththi, James D Ford, Chandni Singh, Carol Zavaleta-Cortijo, Keith Hyams, J Jaime Miranda, Ingrid Arotoma-Rojas, Cecil Togarepi, Harpreet Kaur, Jasmitha Arvind, Halena Scanlon, Didacus B Namanya, Cecilia Anza-Ramirez, COVID- Observatories Team

**Affiliations:** 1 University of the Sunshine Coast, Sippy Downs, Queensland, Australia; 2 Department of Geography, Virginia Polytechnic Institute and State University, Blacksburg, VA, United States of America; 3 Priestley International Centre for Climate, University of Leeds, Leeds, United Kingdom; 4 School of Environment and Development, Indian Institute for Human Settlements, Bangalore, India; 5 Unidad de Ciudadanía Intercultural y Salud Indígena (UCISI), Facultad de Salud Pública y Administración, Universidad Peruana Cayetano Heredia, Lima, Peru; 6 Department of Politics and International Studies, University of Warwick, Coventry, United Kingdom; 7 CRONICAS Center of Excellence in Chronic Diseases, Universidad Peruana Cayetano Heredia, Lima, Peru; 8 Department of Animal Production, Agribusiness and Economics, School of Agriculture and Fisheries Sciences, University of Namibia, Windhoek, Namibia; 9 Indian Institute for Human Settlements, Bangalore, India; 10 Ministry of Health, Uganda National Health Research Organisation, Entebbe, Uganda

**Keywords:** COVID-19, SARS-CoV-2, Indigenous peoples, first nations, aboriginal peoples, American Indians, ethnic minorities

## Abstract

Past influenza pandemics including the Spanish flu and H1N1 have disproportionately affected Indigenous Peoples. We conducted a systematic scoping review to provide an overview of the state of understanding of the experience of Indigenous peoples during the first 18 months of the COVID-19 pandemic, in doing so we capture the state of knowledge available to governments and decision makers for addressing the needs of Indigenous peoples in these early months of the pandemic. We addressed three questions: (a) How is COVID-19 impacting the health and livelihoods of Indigenous peoples, (b) What system level challenges are Indigenous peoples experiencing, (c) How are Indigenous peoples responding? We searched Web of Science, Scopus, and PubMed databases and UN organization websites for publications about Indigenous peoples and COVID-19. Results were analyzed using descriptive statistics and content analysis. A total of 153 publications were included: 140 peer-reviewed articles and 13 from UN organizations. Editorial/commentaries were the most (43%) frequent type of publication. Analysis identified Indigenous peoples from 19 different countries, although 56% of publications were centered upon those in Brazil, United States, and Canada. The majority (90%) of articles focused upon the general adult population, few (<2%) used a gender lens. A small number of articles documented COVID-19 testing (0.04%), incidence (18%), or mortality (16%). Five themes of system level challenges affecting exposure and livelihoods evolved: ecological, poverty, communication, education and health care services. Responses were formal and informal strategies from governments, Indigenous organizations and communities. A lack of ethnically disaggregated health data and a gender lens are constraining our knowledge, which is clustered around a limited number of Indigenous peoples in mostly high-income countries. Many Indigenous peoples have autonomously implemented their own coping strategies while government responses have been largely reactive and inadequate. To ‘build back better’ we must address these knowledge gaps.

## 1. Introduction

COVID-19 has impacted health and well-being globally. As of 1 October 2022, over 220 countries and territories have had cases of COVID-19, while official figures show more than 615 million people have been infected and more than 6.54 million have died the World Health Organization (WHO) estimate the total mortality related to COVID-19 for 2020 and 2021 is around 14.9 million (WHO 2022a, 2022b). Historically Indigenous peoples have suffered disproportionately during influenza pandemics like COVID-19. For instance, the 1918 influenza pandemic saw death rates seven times higher for Māori than non-Māori in New Zealand (McLeod *et al* 2020); in western India, the Adivasis population reduced by 5.4% while the non-Adivasis increased (Hardiman 2012). More recently, the 2009 H1N1 influenza pandemic saw death rates for Indigenous peoples in the Amazon, 4.5 times higher than Brazil’s general population (Ruche *et al* 2009). In the United States (US), the fatality of American Indians/Alaskan Natives was four times higher than the non-Indigenous population (Centers for Disease Control and Prevention 2009). Meanwhile, in Canada First Nations were three times more likely to be hospitalized and six times more likely to be admitted to intensive care than the non-First Nations population (Boggild *et al* 2011). The same pattern was repeated in Australia with Aboriginal Australians experiencing higher rates of hospitalization (Flint *et al* 2010). This is cause for concern given the shorter duration of these pandemics compared to COVID-19. Since the WHO declared COVID-19 a pandemic on 11 March 2020 there have seven outbreak waves and numerous publications. This review provides an overview of the state of understanding of the experience of Indigenous peoples for the first 18 months. It is illustrative of the state of knowledge available to governments and decision makers for addressing the needs of Indigenous peoples in these early and most vulnerable months of the pandemic.

Several factors can increase the risk of Indigenous peoples to COVID-19. Globally, life expectancy is up to 20 years lower for Indigenous peoples than their non-Indigenous counterparts (Anderson *et al* 2016). They also experience greater premature births and infant mortality rates (Chen *et al* 2015). There is a high incidence of infectious diseases including respiratory infections, tuberculosis, and zoonotic diseases (Gracey and King 2009). At the same time, they experience a high burden of malnutrition and non-communicable diseases, such as type 2 diabetes, obesity, hypertension, and heart disease (Yu and Zinman 2007, Anderson *et al* 2016). Together these conditions increase the risk of contracting COVID-19 (Liu *et al* 2020a).

The health challenges faced by Indigenous peoples are linked to the social and physical environments in which they live. The poor determinants of health experienced by Indigenous peoples, including low income; poor access to formal education; high unemployment; inadequate access to safe drinking water, sanitation, and health care; high food insecurity; poor housing; lack of adequate and contextual formal safety nets; and stigmatization, undermine their health (Gracey and King 2009, Anderson *et al* 2016). Additionally, the oppressive legacy of colonization has restrained Indigenous values, culture, and spiritual practices, further reducing the health and well-being of individuals and communities (Valeggia and Snodgrass 2015). Meanwhile, many live in areas experiencing climate extremes and long-term climate change that increase the risk of COVID-19 of transmission (Rahman *et al* 2021, Ford *et al* 2022).

Indigenous peoples have been identified as particularly vulnerable to COVID-19. Yet they are often combined with other marginalized groups such as African Americans, which negates the spiritual and cultural connections they have to the environment that can be a source of vulnerability and resilience. However, there is limited information on the impacts the pandemic is having and how they are responding (Zavaleta-Cortijo *et al* 2020, FAO 2021). We begin addressing this gap by examining three questions: (a) How is COVID-19 impacting the health and livelihoods of Indigenous peoples, (b) What challenges are Indigenous peoples experiencing, (c) How are Indigenous peoples responding? Answering these questions allows scientists to generate feedback to improve vaccination uptake and post COVID development in Indigenous communities. In addressing these questions this systematic scoping review identifies key knowledge trends, clusters, and gaps, and proposes recommendations for future research. This paper provides a broad overarching view of the scholarship that focuses on Indigenous peoples and COVID-19 through the first 18 months of the pandemic. It does not provide an epidemiological or meta-analysis of health, vaccinations, or socio-economic impacts.

## 2. Methods

Systematic scoping reviews employ a systematic methodology to provide an overview of the literature upon a topic (Levac *et al* 2010). Unlike a systematic review which focuses upon a narrow topic, a scoping review addresses broader questions and is suitable for emerging issues, such as the experience of Indigenous peoples during COVID-19 (Berrang-Ford *et al* 2015). The three research questions build upon our previous framework that analyses Indigenous people’s resilience to emergent socioenvironmental threats (Ford *et al* 2020, Galappaththi *et al* 2020).

### 2.1. Defining indigenous peoples

There is no universal definition of Indigenous peoples. The concept of Indigenous peoples emerged in response to the process of colonization by European forces that saw the subjugation of some, but not all aboriginal peoples (Secretariat of the Permanent Forum on Indigenous Issues 2009). The United Nations Permanent Forum on Indigenous Issues provides the following criteria, that we used to determine ‘Indigenous’: ‘identify themselves and are recognized and accepted by their communities as indigenous; demonstrate historical continuity with pre-colonial and or pre-settler societies; have strong links to territories and surrounding natural resources; have distinct social, economic or political systems; maintain distinct languages, cultures and beliefs, form non-dominant groups of society, resolve to maintain and reproduce their ancestral environments and systems as distinctive peoples and communities’ (Secretariat of the Permanent Forum on Indigenous Issues 2009).

### 2.2. Search strategy and selection criteria

The COVID-19 pandemic is a rapidly evolving situation with articles and evidence being published in peer reviewed journals and by organizations including the United Nations. Hence this review includes grey literature from UN agencies. Relevant publications for this review were identified by using a systematic search strategy for electronic databases and by manually searching select UN agency websites. The strategy (S1 table) was applied to Web of Science, Scopus, PubMed databases and UN agencies. All databases and UN agencies were searched on 1 March 2021, to identify publications from 1 January 2020 to 31 January 2021; then again on 4 July 2021 from 1 February 2021 to 4 July 2021. The inclusion and exclusion criteria employed are shown in table 1.

**Table 1.** Inclusion and exclusion criteria applied to the results of the search in databases and UN agencies.

Inclusion	Exclusion
All languages	No Language
Articles published between 1 January 2020 and 4 July 2021	Articles published before 1 January 2020
Peer reviewed journal articles including original research, editorials, commentaries, essays, and reports, policy statements published by UN organizations	Conference abstracts or proceedings, newspaper articles, books, chapters, blogs and reports by non-UN organizations
Articles directly and significantly focus on an Indigenous population, community or group as defined by United Nations Permanent Forum on Indigenous Issues	Articles that do not directly and significantly focus upon an Indigenous population, community or group as defined by United Nations Permanent Forum on Indigenous Issues
Articles that directly and significantly focus on the COVID-19 virus or the COVID-19 pandemic	Articles that are do not directly and significantly focus upon COVID-19 virus or the COVID-19 pandemic
Evidence includes systematic reviews, meta-analysis, random controlled trials, quasi-experimental, cohort studies, descriptive studies, qualitative studies, literature reviews, opinion of authorities and reports from expert committees	None

**Figure 1.**
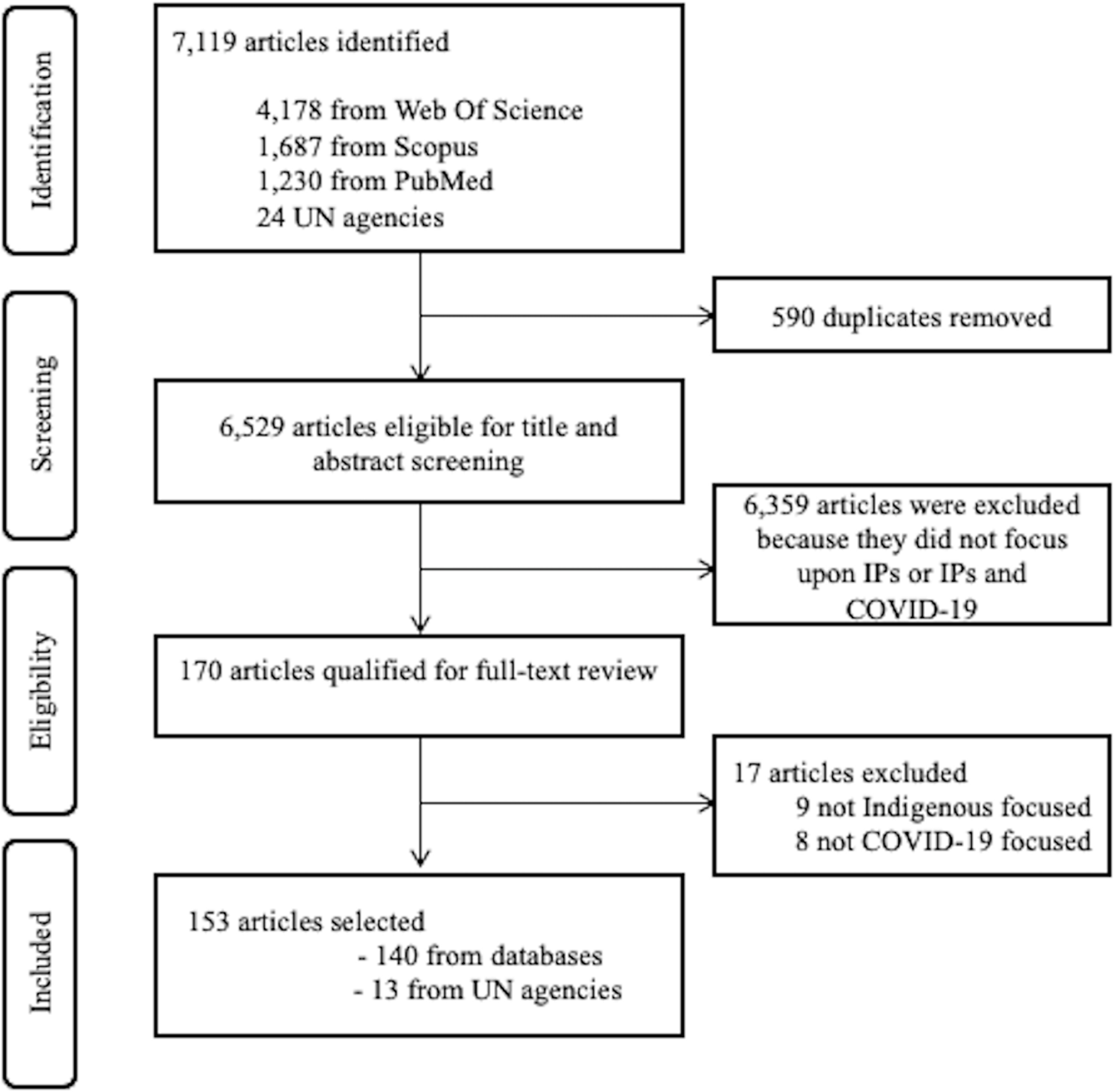
PRISMA diagram of selection process for the scoping review of COVID-19 and indigenous peophles articles and results 1 January 2020–4 July 2021.

Our search identified a total of 7119 publications between 1 January 2020 and 4 July 2021 in the mentioned databases and UN agencies. The titles and abstracts were exported to Microsoft Excel and 590 duplicates were removed. Two reviewers, who had completed a training workshop on implementing the screening protocol and had completed two blind trials of screening papers with a 90% agreement, independently screened titles and abstracts to determine which publications would be included. Any disagreements or discrepancies were resolved by discussion between the reviewers. A total of 6359 articles were excluded because the focus was not on Indigenous peoples and COVID-19. The remaining 170 publications were downloaded for a full text screening conducted by four reviewers (who are co-authors based in Canada, Australia, and India). A further 17 articles were removed: Indigenous peoples were not the focus in nine and COVID-19 was not the focus in eight. In total 153 publications (140 peer-reviewed articles and 13 from UN agencies) were retained for final review (figure 1). Additional quality control occurred via bi-weekly meeting with Kerrie Pickering (KP) and Eranga Galappaththi (EG) to verify steps and process. Monthly progress meetings were held with all the authors for high level feedback. The unit of analysis was each article, the quality of each article was not assessed.

From our initial analysis four themes evolved: COVID-19 virus and Indigenous peoples; impacts of pandemic restrictions on livelihoods and food security; system level challenges; responses by Indigenous peoples, government and organizations. Within these four themes, we designed 12 coding questions to answer the research questions (S2 table). The answers to the coding questions were analyzed using content analysis (Downe-Wamboldt 1992). Themes that emerged from the content analysis were: testing/incidence/hospitalization/mortality of COVID-19 in Indigenous peoples compared to the non-Indigenous population and pre-existing health conditions; effects of pandemic restrictions upon cultural practices, livelihoods and income; social and ecological challenges; government, organization, community and cultural responses. Microsoft Excel 2019 was used for descriptive statistics.

## 3. Results

### 3.1. Descriptive results

**Figure 2.**
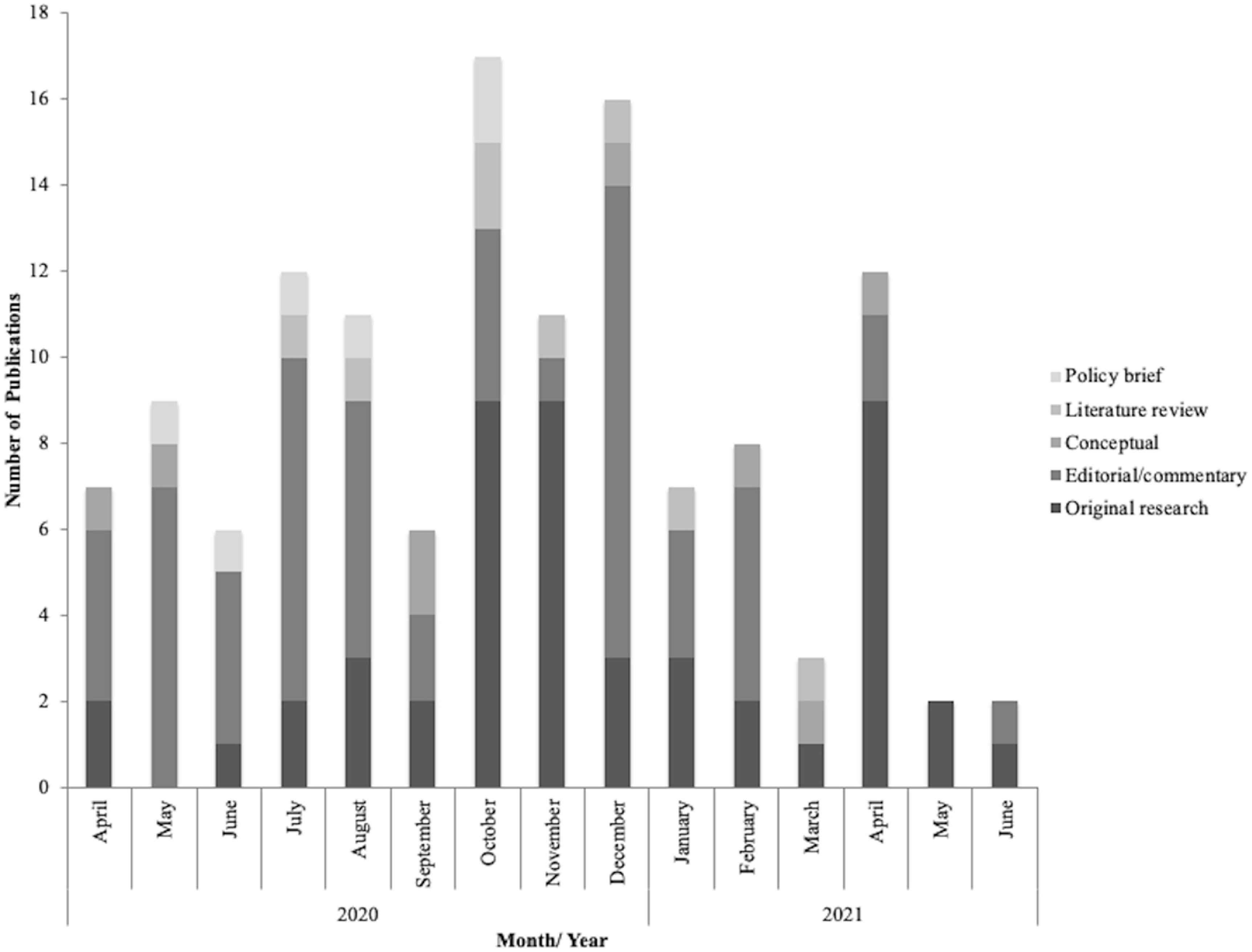
Number of publications by type, month, and year. Of the 153 articles 130 identified a single publication month, the graph displays the results from those articles. The graph begins when the first articles were published, April 2020. July 2021 is not included as the online search was on 4 July 2021 and did not capture the entire month of July.

Our study identified 153 publications discussing Indigenous peoples and COVID-19 from 1 January 2020 to 4 July 2021 of which, 140 were peer reviewed and 13 were grey literature from UN agencies (S3 Table). The pandemic was declared on 11 March 2020 and three weeks later on 1 April, UN Women (2020) released a report and on 7 April the journal Nature published the first article, a correspondence by Zavaleta (2020) on the pre-existing lack of Indigenous peoples data. The peer reviewed publications were spread across 101 unique journals, most (95%) of the journals published one or two articles. The three journals with the greatest publications were Morbidity and Mortality Weekly Report (*n* = 8), Alternative—An International Journal of Indigenous Peoples (*n* = 7), and Mundo Amazonico (*n*= 5). The most (43%) frequent type of articles were editorial/commentaries followed by original research (38%) with fewer publications being conceptual (7%), literature reviews (6%) or policy briefs (6%) (figure 2). The majority (80%) of editorials were published in 2020, while original research remained constant at three publications per month since April 2020. In 2020, there was an average of 10 publications (of all types) per month. The greatest publications were in October 2020 (*n* = 17), then December (*n* = 16). In 2021, publications dropped to an average of six per month.

**Figure 3.**
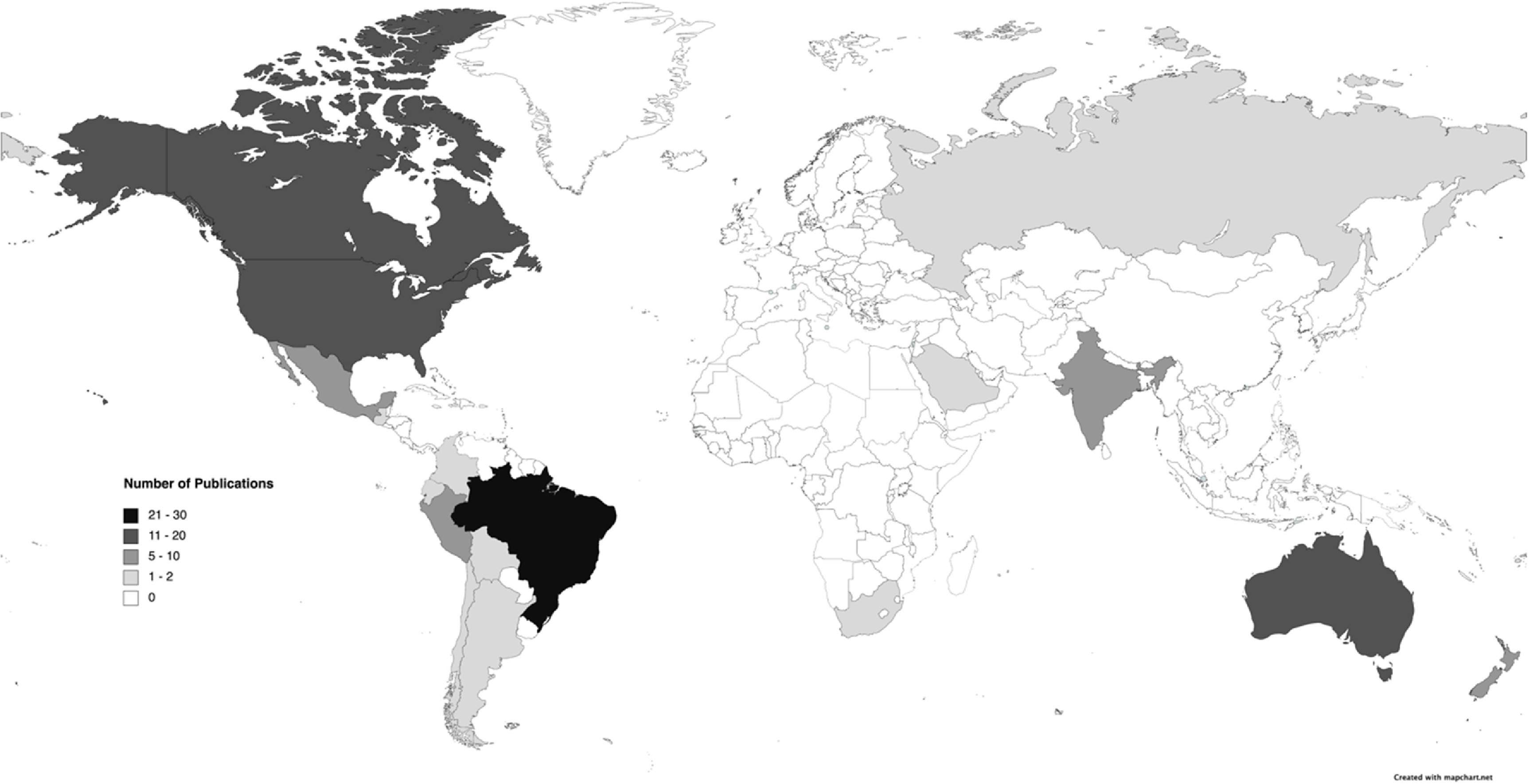
Country of Indigenous Peoples and the number of publications per country. The most publications were in Brazil including the Korubo, Xikrin Yanomami, and the Quilombolas, followed by the United States, Canada, and Australia. White indicates an absence of publications. Map created with mapchart.net.

Of the peer reviewed articles, the three countries with the highest first author affiliation were the US 29% (*n* = 40), Brazil 18% (*n* = 25), and Canada 15% (*n* = 21). Twenty-two per cent of authors identified receiving funding, this was largely (79%) by government agencies, the US National Institute of Health, funded most (29%) of these publications. Analysis identified publications focused on Indigenous peoples from 19 different countries (figure 3). The most (23%) articles focused on Indigenous peoples in Brazil, then American Indians/Alaska Natives (17%) in the US, followed by First Nations, Inuit and Métis (16%) in Canada. The majority (90%) of articles focused on the general adult population. Four articles looked at youth, while two applied a gender lens, demonstrating a gap in how the pandemic has had gendered and intersectional impacts. Of the peer reviewed articles, 29% had authors who self-identified as Indigenous. For the grey literature, six UN agencies had publications, Department of Economic and Social Affairs (DESA), Food and Agriculture Organization (FAO), International Labor Organization (ILO), Pan American Health Organization (PAHO), WHO, United Nations Children’s Fund (UNICEF) and UN Women. The PAHO/WHO had the most (*n* = 4) publications followed by ILO (*n* = 3) then UNICEF (*n* = 2).

### 3.2. Impacts of COVID-19 on the health and livelihoods of Indigenous peoples

To answer the first research question on how COVID-19 is affecting Indigenous peoples, we split the question into two separate (but interacting) themes: the COVID-19 virus and pandemic restrictions. The next two sections discuss these themes sequentially.

#### 3.2.1. COVID-19 virus and Indigenous peoples

We analyzed the interaction between Indigenous peoples health and the COVID-19 virus through reported testing rates, incidence, hospitalizations, and mortality. Our analysis showed a lack of access to ethnically disaggregated data and incomplete data collected were major barriers to understanding the impact COVID-19 was having on Indigenous peoples health (Banning 2020b, Canalez *et al* 2020, Curtice and Choo 2020, Department of Economic and Social Affairs 2020, p 70, Follent *et al* 2021, Lavoie *et al* 2020, Menton *et al* 2021, Power *et al* 2020). Testing for COVID-19 provides a crucial tool in preventing outbreaks (Morales-Narváez and Dincer 2020). We found very few (0.04%) articles documented testing rates for Indigenous peoples, which may suggest that they are largely unknown. In the US, Canada, and Ecuador testing rates for Indigenous peoples were below the national average, reasons for this included a lack of testing kits (Crooks *et al* 2020, Eades *et al* 2020), the remoteness of some communities challenged the transportation of kits (Bogdanova *et al* 2020, Burki 2021), and people refusing to be tested due to a lack of trust in their respective government (Jenkins *et al* 2020, Kerrigan *et al* 2021, Matthias *et al* 2021, Ortiz-Prado *et al* 2021). Rodriguez-Lonebear *et al* (2020) argues the lack of testing availability in the Navajo Nation, US, resulted in uncontrolled outbreaks causing it to be labeled as the an epicenter of the virus.

Few publications reported the incidence (18%) or mortality (16%) from COVID-19 amongst Indigenous peoples. Of these publications, there was no clear trend. Some countries identified a greater COVID-19 incidence and mortality amongst Indigenous peoples. In Brazil, Fellows *et al* (2021) found COVID-19 incidence was 136% higher and mortality 110% higher amongst Indigenous peoples when compared to the national average. Similarly in Montana, US, American Indians/Alaska Natives experienced greater COVID-19 incidence (2.2 times) and COVID-19 mortality (3.8 times) than the White population (Williamson *et al* 2021). While in Chile, Millalen *et al* (2020) used regression modeling on aggregated data finding municipalities with higher percentages of Indigenous peoples experienced higher COVID-19 infection and mortality rates, suggesting Indigenous peoples create greater vulnerability to COVID-19. In contrast, lower infection rates were documented in Aboriginal and Torre Strait Islanders, Australia, (0.8% of the national population (Eades *et al* 2020, Moodie *et al* 2020)) and in Canada for First Nations living on reserves, incidence was one-quarter and fatality rates a fifth (Banning 2020b).

Underlying health conditions increase the risk of contracting COVID-19 (CDC 2021). Pre-existing health conditions were identified as a concern in 49% of the reviewed publications. Communicable diseases including malaria, tuberculosis, and human immunodeficiency virus (HIV)/acquired immunodeficiency syndrome (AIDS) were cited most (58%) often. Across Africa the high burden of disease from HIV is expected to have wide-ranging impacts for COVID-19 (Dune *et al* 2020). Slightly fewer articles (55%) identified non-communicable diseases including obesity and diabetes, while just under half (48%) identified malnutrition/under-nutrition. The negative impacts of the pandemic restrictions upon mental health have also been noted (Pfefferbaum and North 2020). Several (12%) articles drew attention to mental health disparities Indigenous peoples experience, including greater rates of suicide, addiction, self-harm (Moodie *et al* 2020, Usher *et al* 2020, Graves *et al* 2021) and the psychological impacts of intergenerational trauma (John-Henderson and Ginty 2020), warning the pandemic may further undermine mental health.

#### 3.2.2. Impacts of pandemic restrictions on livelihoods and food security

The second theme in our analysis was to explore the impact of the pandemic restrictions upon Indigenous peoples. The variable nature of the pandemic over time and spatial scales makes a simple analysis of pandemic restrictions challenging. To different degrees and during different waves, governments across the globe enforced restrictions such as lockdowns and stay at home orders to limit the spread of the COVID-19 virus. The effectiveness and compliance with such response policies has been called into question as they have caused substantial economic and social costs and increased mental health challenges for many individuals (Onyeaka *et al* 2021). These restrictions have had multiple, differentiated impacts on Indigenous peoples. Movement restrictions have restrained foraging for food for the Batwa in Uganda, (Zavaleta-Cortijo *et al* 2020), Qom/Toba people in Argentina (Haas *et al* 2021), Terena communities in Brazil (Ribeiro and Rossi 2020), and hunter gatherers in the jungles of Borneo (FAO 2021). In East Africa these restrictions have intensified the stressors for nomadic tribes to respond to recent desert locust outbreaks (FAO 2021).

Meanwhile the sudden closure of businesses has reduced employment for many Indigenous migrants forcing them to return to their home villages and leaving them and their families without an income (ILO 2020, Cohen and Mata-Sánchez 2021, Kasi and Saha 2021, Saxena *et al* 2020). In India, the rapid lockdown measures disrupted public transportation forcing many people to walk long distances to get back to their villages and hampered access to local markets to buy and sell food (Saxena *et al* 2020). Kasi and Saha (2021) report in the Dindigul district of Tamil Nadu, India movement restrictions prevented tribal people from selling goods such as tamarind, wild honey and sal leaves at markets, their main source of income. In Northern Russia, Potravnaya and Sleptcov (2020) document that the loss of tourism has reduced demand for local crafts, decreasing the income of many reindeer herders. Meanwhile, Bogdanova *et al* (2020) found costs for these same herders increased as they were not allowed to enter cities where they routinely purchase prophylactic medications for their herds, instead they were forced to rely upon trading posts that had higher prices and less supply.

Transportation and movement restrictions have also impacted the flow of goods and services into communities, affecting food security. In the Pacific Island Region, Davila *et al* (2021) documented disrupted sea transportation caused a lack of agricultural items including seeds, equipment, and fertilizer delaying planting for many small-holder farmers. In North Western Ontario, Canada, Levkoe *et al* (2021) identified delays in transportation caused produce to be spoiled reducing the quality and quantity of available food, while food banks were forced to close further reducing food availability. Meanwhile, Hoover (2020) explains in the US over half of American Indian/Alaska Native children rely on school lunch programs and their food insecurity has intensified with the sudden closure of these programs. However, in India fear of contracting COVID-19 from vendors has caused some villages to stop vendors coming, reducing food availability (Saxena *et al* 2020).

### 3.3. Systems level challenges

Pre-existing structural inequities mediated Indigenous peoples capacity to respond to the COVID-19 virus and pandemic restrictions. Our second research question sought to identify these challenges. Five types of challenges emerged from our analysis: ecological, poverty, communication, education and health care services. Ecological challenges were the least prominent, identified by 39% of articles. Most (33%) articles identified a lack of adequate water and sanitation prevented hand hygiene, a first line of defense in the transmission of many other diseases that can make people more susceptible to contracting COVID-19. Other ecological challenges included deforestation (11%), followed by climate change (6%) then pollution (3%). Authors documented ecological factors are compounding other structural factors potentially increasing risks of COVID-19. For example, de León-martínez *et al* (2020) explains in Mexico many Indigenous households use solid fuel indoor cooking stoves which reduce indoor air quality and are associated with pulmonary diseases such as lung cancer, and acute respiratory infections, making these populations more susceptible to contracting COVID-19. At a broader scale, Haas *et al* (2021) identify that in Chaco Province, Argentina, widespread deforestation has altered the local climate and driven water scarcity, making actives such as wild food gathering (e.g. picking berries, hunting) and hand-washing more difficult.

A range of social challenges were identified in 59% of the reviewed documents. Of these articles, the impacts of factors linked to structural inequity from colonialism, racism, loss of land rights, and violations of human rights were cited most frequently (93%). Racism caused some Indigenous peoples to be stigmatized as sources of COVID-19 causing severe movement restrictions to be enforced. For example, Haas *et al* (2021) describe during an outbreak in May 2020, lasting several weeks the Gran Toba, in Chaco Province, Argentina, were singled out by the regional government, who physically isolated the settlement with earth barriers and fences preventing people and goods from entering and leaving the community. In Brazil, Menton *et al* (2021) reports the mayor of Pau D’Arco decreed a lockdown to prevent members of a nearby village from entering the city, yet many Indigenous peoples in Brazil are increasingly being exposed to COVID-19 as they encounter individuals involved in illegal mining and land grabbing actions on their traditional lands.

Poverty, employment and overcrowding were reported as significant challenges as well. Indigenous peoples are three times more likely to live in extreme poverty compared to non-Indigenous people (ILO 2020). Poverty was most often (57%) identified as increasing the incidence and poor outcomes of COVID-19, across the Americas, Africa, India and Asia, and was associated with food insecurity and malnutrition (FAO 2021, Hoover 2020, Leite *et al* 2020, Rodriguez-Lonebear *et al* 2020, Teixeira 2020). One of the most important ways in which poverty increases vulnerability to COVID-19 is that it requires people to go out to work increasing their risk of exposure. Globally, more than 86% of Indigenous peoples work in the informal sector where the low pay and lack of social protection has forced them to keep working (ILO 2020). Those in the formal sector are largely in service occupations that further increase their risk of exposure such as domestic work, health care, transportation and construction (ILO 2020). COVID-19 spreads quickly when people are close together. Overcrowding due to inadequate housing was found to be a challenge for many (43%) Indigenous peoples.

A lack of communication strategies about COVID-19 prevention in Indigenous languages that were also culturally appropriate was another challenge identified by several articles (9%). To improve access to information about COVID-19, some governments have translated information into local languages and made culturally appropriate flyers. The FAO (2020) notes in Columbia the government issued COVID-19 messages in Indigenous languages and in Peru the government developed informational posters and radio spots. In New Zealand, Māori organizations collaborated to create their own culturally appropriate flyer (Te One and Clifford 2021). Digital platforms have also been useful for communicating health information. For example, in Gran Toba, Argentina members of a bilingual school created and shared a digital video in three Indigenous languages (Haas *et al* 2021). Additionally, social media platforms have provided a tool for activism by youth and young adults to increase local and global awareness of the struggles Indigenous peoples are facing. For example, in Brazil Franco and da Silva (2021) describe the use of Instagram by Indigenous peoples to create awareness of Indigenous rights violations and to mobilize organized forms of resistance. Menton *et al* (2021) explains in Brazil social media platforms have enabled Indigenous peoples to access crowdfunding to support their efforts to take the government to court for a lack of support. However, Haas *et al* (2021) also notes these platforms have been used as a vehicle for hate speech against Indigenous peoples. Communication strategies will need further evaluation to understand the effectiveness and cultural adequacy in preventing COVID-19 and or promoting vaccination among Indigenous peoples.

A challenge for many Indigenous children and youth has been the disruption to their education. Many countries have taken classes online and as UNICEF (2020) explains, this disadvantages Indigenous children and youth, as many communities lack digital coverage, equipment, technical expertise, and programming in their languages. For example, in Mexico, 54% of Indigenous children (7–17 years) lack access to a radio.(UNICEF 2020). Beyond the logistics, Dietz and Cortés (2021) raise concerns that in Mexico the return to a Spanish only curriculum will undo the years of work to include Indigenous languages and cultures with the goal of making education more relevant to students and keeping them attending school longer. However, the closure of schools has been beneficial for some groups providing an opportunity to recover indigenous knowledges. For example, in Canada, Neeganagwedgin (2020) describes how some First Nations children are now being taught on the land by elders. While in northern Russia, Potravnaya and Sleptcov (2020) found the closure of schools allowed reindeer farmers to take their children with them to summer pastures.

The prevention and treatment of COVID-19 require access and trust in health care services. Yet, across all 19 countries, authors (37%) identified inadequate access to health care was a chronic challenge for Indigenous peoples. For example, Ferrante and Fearnside (2020) explain in Brazil the lack of access to health care has intensified, as prior to the pandemic, the Bolsonaro administration had dismissed 8000 doctors, who served mostly Indigenous peoples in the interior. Authors (Curtice and Choo 2020, Teixeira 2020, Mosby and Swidrovich 2021, Pratt *et al* 2021, Speers 2021) have stressed given their greater risk of contracting COVID-19 Indigenous peoples need to be prioritized for early vaccination. However, other authors caution due to past involuntary medical treatment and experimentation, many Indigenous peoples may be hesitant to be vaccinated. For example Mosby and Swidrovich (2021) explain in Canada some First Nations, Métis and Inuit elders remember having vaccines tested on them as children at residential school and are weary of the new vaccines. Adding to this concern is the federal governments prioritizing of First Nations, Métis and Inuit for vaccinations, with some feeling these populations were again being used to test these new vaccines (Mosby and Swidrovich 2021).

### 3.4. Responses by governments, Indigenous peoples and organizations

Our analysis identified responses to the pandemic are being generated from two types of actors: national and sub-national governments and Indigenous peoples themselves at the individual, community and organizational scale. We found governments were implementing a range of short- and longer-term, largely reactive responses. Government in Ecuador (Tuaza Castro 2020), India (Kasi and Saha 2021), Peru (Reinders *et al* 2020), and Bolivia (Kaplan *et al* 2020), provided intermittent food rations and vouchers to those in need when food supplies became limited, although in some regions those receiving government assistance prior to the pandemic were excluded from these initiatives (Reinders *et al* 2020). Other governments chose to bolster current programs with additional funding over a period. For example, the US federal government provided an additional US$2.4B to the Indian Health Service (Burki 2021) and US$100 M to the Food Distribution Program on Indian Reservations (Hoover 2020). While the Australian government chose to direct US$11 M toward Indigenous suicide prevention (Walker *et al* 2021). In contrast to these programs, the federal government of Canada gave funds directly to communities to use as they saw fit (Hillier *et al* 2020). In response to lesson learnt from the H1N1 pandemic, the Australian Government also took a pre-emptive approach, convening the Aboriginal and Torres Strait Islander Advisory Group on COVID-19 soon after the pandemic was declared, to ensure the Indigenous population were actively involved in all phases of national planning (Crooks *et al* 2020). However, not all government assistance came willingly; federal governments in the US (Yellow Horse and Huyser 2021) and Brazil (Charlier and Varison 2020) were ordered by their respective supreme courts to provide relief funds for Indigenous peoples. Overall, during the first 18 months it was governments who were working with Indigenous population prior to the pandemic that implemented policies that were most helpful.

There is also significant evidence to suggest that many Indigenous communities and organizations proactively took action to protect their communities. Roadblocks and checkpoints were most (31%) frequently used to prevent people from entering the communities and to assess for symptoms of COVID-19 (Kaplan *et al* 2020, Goha *et al* 2021, Menton *et al* 2021). Although the Yanomami, Brazil chose to isolated by moving into the forest (Goncalves *et al* 2020). In several Pacific Island communities, mandatory quarantine areas were set up for those returning to the community (Davila *et al* 2021). However, isolation measures were not always successful, Ortiz-Prado *et al* (2021) show in the Amazon regions of Ecuador such isolation measures failed to contain the spread of COVID-19.

Isolation measures slowed the flow of goods and services into a community. To ensure people had adequate access to water, food and medication during the isolation periods, some communities built upon existing networks and programs. For example, in New Zealand Māori organizations Te Pūtahitanga and Whānau Ora navigators collaborated to respond to lockdown needs of Māori by supplying food and hygiene packs, grants for home heating and digital devices (McMeeking *et al* 2020). In North Western Ontario, Canada, the Red Rock Indian Band began ordering directly from bulk food suppliers and partnered with local businesses to get a constant supply of fresh fruit and vegetables (Levkoe *et al* 2021). Others, including First Nations in Canada (Banning 2020a) and the US (Hoover 2020) returned to traditional communal practices of sharing and caring for one another. To boost the local food supply in Pacific Island communities, farmers focused on producing local foods instead of cash crops (Davila *et al* 2021). Similarly, in Mexico Indigenous Oaxaca, increasingly sourced food from their home gardens (Cohen and Mata-Sánchez 2021).

Other responses by Indigenous peoples included using Indigenous knowledge and medicines, as identified in 13% of the articles. For example, da Silva *et al* (2021) found in the Alto Rio Solimões Special Indigenous Sanitary District, over half (54%) of the patients who attended a medical clinic with symptoms of COVID-19 had sought treatment using local Indigenous medicines. Similarly, in Brazil, Mondardo (2020) identified Indigenous herbal preparations that were used to treat COVID-19 symptoms. However, this traditional medicine was not always welcomed. Beyers (2020) explains in South Africa, although traditional healers are legally recognised the government has not included them in strategies to manage the pandemic.

## 4. Discussion

In this scoping review, we provide a broad overview of the peer reviewed literature from three databases and the grey literature from UN agencies focused on Indigenous peoples and COVID-19 in the first 18 months of the COVID-19 pandemic. Our analysis identified an overall lack of knowledge on the impact COVID-19 is having on Indigenous peoples. The wide range of journals (101) with few (1–2) publications suggests the experience of Indigenous peoples is an issue of only occasional interest. Although Indigenous peoples make up five of the global population and are spread across 90 countries our review identified publications from only 18 countries, indicating the situation for Indigenous peoples in many regions is unknown and severely understudied (FAO 2020).

The knowledge we do have is clustered around several, mostly high-income countries. Our findings show Brazil, US, Canada, and Australia had the greatest number of first author institutions and the most publications. The lack of coverage from other regions is a concern as our knowledge is dominated by the experience of a few Indigenous peoples and communities yet responses have varied between countries and regions. For example, in the Pacific Island Region countries contained the spread of the virus by implementing lockdowns, providing social safety nets, and setting up mandatory quarantine facilities (Davila *et al* 2021). However, lockdowns were ineffective in many African countries where the majority of people live below the poverty line and economic assistance was not provided, forcing people to go out (Ngepah 2021). The diversity among Indigenous cultures and practices is well known and caution must be taken not to generalize the experience of these communities as representing all Indigenous peoples.

Additionally, we identified a deficit on how Indigenous women and children are experiencing COVID-19. Research has shown the COVID-19 pandemic has disproportionately impacted women, children, and youth who face greater exposure and are at greater risk. Pregnant women are among those most vulnerable (Liu *et al* 2020b, Danianto *et al* 2021, Godleski *et al* 2022). Women make up the majority of health care workers, they are often the primary caregivers in their households, and are more likely to live in poverty and experience domestic violence; however we found few articles within Indigenous populations (Wenham *et al* 2020). Articles we did find either focused on reproductive health or took a broad perspective such as UN Women, to guide policy makers and bring awareness that women were a vulnerable population (UN Women 2020). The plight of women was mentioned in passing in several articles within the context of the broader Indigenous population. For example, Kasi and Saha (2021) and ILO (2020) report women are more vulnerable to sexual violence during lockdown periods. In contrast Akuhata-Huntington *et al* (2020) and Argumedo *et al* (2020) draw attention to the important role women play in holding and sharing traditional knowledge, making them a source of resilience during and post pandemic. Considering the extremes of these reports there is a need to apply a gender and intersectionality lens (Sultana 2021) to better understand the experience of Indigenous women during COVID-19. Additionally applying a sex lens would enable a greater understanding through different age groups and the experience of men.

Should the decrease in publications continue it is likely the current knowledge gaps and clusters will continue. This review shows a reduction in the average monthly number of publications from 10 in 2020 to six in 2021. This is largely in response to a reduction in the number of editorials in 2021. Singh and Singh (2006) explain journal editorials serve an important role as brief articles that ‘tackle recent events and issues and attempts to formulate viewpoints based on an objective analysis of happening and conflicting/contrary opinions’ (p 15). It is not unreasonable to speculate that early in the pandemic, scholars wrote editorials as an expedient means to communicate the challenges Indigenous peoples face to inform decision makers as policies and guidelines were being developed. However, as the editorials have declined there has not been an increase in other types of publication. Indeed, this review identified few (6%) documents focused on policy briefings implying there is limited guidance for countries on how to act in an Indigenous peoples context.

This systematic review sought to answer three research questions, the first, how is COVID-19 impacting the health and livelihoods of Indigenous peoples? We found multiple authors identified a lack of health data disaggregated by ethnicity to be preventing statistical analyses to determine the incidence and mortality from COVID-19 and the factors and pathways that influence the susceptibility of Indigenous peoples to the virus. The incidence and mortality of COVID-19 for Indigenous peoples varied; in Brazil and the US Indigenous peoples experienced greater occurrence than the general population but in Australia and Canada it was lower. The PAHO and the WHO have repeatedly requested that governments collect ethnicity data alongside health data (PAHO and WHO 2020). The lack of health data about Indigenous peoples is not a new problem, and further studies are needed on ways this data can be collected and managed where gaps do exist.

Pandemic restrictions have negatively impacted livelihoods and food security for Indigenous peoples. Globally, over 86% of Indigenous peoples work in the informal economy and are three times more likely to be in extreme poverty than non-Indigenous people (ILO 2020). We found the sudden loss of income from agriculture, fisheries, and employment has reduced the flow of money and goods into communities and households. Furthermore, over 70% of Indigenous peoples live in rural areas (ILO 2020) where the abrupt cancellation of public transport, which many rely on has created an additional burden to return home and get to markets to buy and sell food and goods. Our findings showed communities reliant on purchasing their food, such as in Northern Ontario, Canada (Levkoe *et al* 2021), and settlements in Australia (Follent *et al* 2021), were more likely to experience food insecurity compared to households who produced some of their own food, such as the Oaxaca in Mexico, who increased their production (Cohen and Mata-Sánchez 2021). However, we found little information on the experience of Indigenous peoples in urban settings and as Indigenous peoples increasingly move to towns and cities there is an increasing need to address this knowledge gap.

Indigenous peoples are experiencing a range of system level challenges affecting their susceptibility to COVID-19. Similar to other studies including Emerson and Montoya, 2021, Lively, 2021, and Modesto *et al* 2022, our analysis also identified the effects of colonization, systemic racism, and structural inequities were shared amongst many Indigenous peoples (ILO 2020). For example, overcrowding and inadequate access to water and sanitation experienced in many Indigenous peoples increased the risk of contracting COVID-19 while a lack of access to health care prevented treatment. However, in countries where there was limited political will to support Indigenous peoples such as Brazil (Fellows *et al* 2021) and the US (Williamson *et al* 2021), outcomes were poorer (higher incidence and mortality) than those in countries such as Australia (Moodie *et al* 2020) and Canada (Banning 2020b) where political support was greater. Hence, context impacts how Indigenous peoples experience COVID-19, if countries such as the US (White House Government 2021) are to ‘build back better’ there is a need for further research on the impacts of government policies on Indigenous peoples during the pandemic (Capano *et al* 2020).

A constant trend through the articles has been the self-autonomy of Indigenous peoples to respond to COVID-19 to maintain their health and wellbeing. Communities have frequently implemented collective culture and practices with younger members utilizing digital technologies. We identified a range of strategies from self-imposed isolation measures (Kaplan *et al* 2020, Goha *et al* 2021, Menton *et al* 2021), communal sharing (Hoover 2020, Rodriguez-Lonebear *et al* 2020) and Indigenous medicines (Mondardo 2020, da Silva *et al* 2021). Additionally, youth and younger adults employed social media campaigns putting the voice of Indigenous peoples and the struggles they are facing to the global audience (Franco and da Silva 2021, Menton *et al* 2021). There is a need to evaluate the effectiveness of such strategies to inform decision makers so they can, where appropriate, incorporate resources to support this autonomy.

Autonomy can also reduce vaccine hesitancy in Indigenous peoples. Vaccines are considered the primary tool to end the COVID-19 pandemic. Although vaccinations did not emerge as a main theme in this systematic review, likely due to the time delay between vaccine rollouts and getting articles published, there is concern that vaccine hesitancy will cause few Indigenous peoples to be vaccinated. Reasons include past medical experimentation, mis-information and a potential lack of culturally appropriate educational materials in Indigenous languages (Hatcher *et al* 2020). Yet some data shows higher vaccination rates for some Indigenous peoples. For example, by September 2021 full vaccination rates for American Indians/Alaska Natives were 8% higher than non-Hispanic White persons (Foxworth *et al* 2021). On 1 February 2022 the Government of Canada reported over 86% of First Nations and Inuit over 12 or older were fully vaccinated compared to the national rate of 79.4% (Canada 2020). The high vaccination rates are partly ascribed to the autonomy of Indigenous peoples to control and provide their vaccinations (Nicholas 2021, Silberner 2021) reinforcing the importance of supporting Indigenous peoples autonomy.

There are limitations in this systematic review. Firstly, the rapidly changing COVID-19 virus, multiple outbreak waves, government responses, vaccination roll outs and time delays in getting articles published means there is a lag in information. For example, although vaccinations are currently a main topic of concern when we did the search, vaccination roll outs had not begun in many countries and there was little evidence of their uptake in Indigenous populations. Different search terms for Indigenous populations may yield more results. Our search of the grey literature relied on UN agencies because we believed these entities would be sharing up-to-date knowledge and insights, to assist governments and organizations. Other sources of grey literature such as government websites, non-government organization (NGO’s), newspapers, and blogs may produce different results. For example, we analyzed the response of governments as reported by authors who may not have documented or been aware of all government strategies to support Indigenous peoples whereas searching government websites for policies may show greater detail. Additionally, government responses have changed over time and with the various pandemic waves, we believe this is a direction for future research.

## 5. Conclusion

This systematic scoping review has analyzed the peer reviewed and grey literature on the experience of Indigenous peoples and COVID-19. The review identified a lack of empirical data which restricts our understanding to a limited number of Indigenous peoples in mostly high-income countries and as publications decrease this knowledge cluster may continue. We found that while Indigenous peoples experience a range of system level challenges, many are autonomously implementing Indigenous knowledge, values, and new strategies such as roadblocks, isolating communities, and social media platforms to protect their communities. However, several important gaps were also identified in the systematic review that need addressing if we are to ‘build back better’. These includes access to ethnically disaggregated data, the application of a gender lens, and a greater exploration of government assistance and autonomous coping strategies communities are implementing.

## Data Availability

All data that support the findings of this study are included within the article (and any supplementary files).

## COVID Observatories tag

Triphine Ainembabazi, Margaret Angula, Amir Bazaz, Lea Berrang-Ford, Joana Bezerra, Victoria Chicmana-Zapata, Shaung Coggins, Frances Crowley Yon Fernandez de Larrinoa, Bhavya George, Martha Hangula, Sherilee Harper, Brianne Jones, Genevieve Jones, Christopher Kazaana, Jyotsna Krishnakumar, Irene Kunamwene, Shuaib Lwasa, Asish Mangalasseri, Clare McGuire, Adelina Mensah, Angus Naylor, Mark New, Nosipho Nkwinti, Richard Nuwagira, Samuel Okware, Maria Osipova, Tristan Pearce, Prathigna Ponnacha Kodira, Bianca van Bavel, Anita Varghese, Carlee Wright
